# Statins in cardiometabolic disease: what makes pitavastatin different?

**DOI:** 10.1186/1475-2840-12-S1-S1

**Published:** 2013-05-30

**Authors:** Henry Ginsberg

**Affiliations:** 1 Irving Institute for Clinical and Translational Research, Columbia University Medical Center, 622 West 168th Street, New York, NY 10032, USA

## Abstract

The term cardiometabolic disease encompasses a range of lifestyle-related conditions, including Metabolic syndrome (MetS) and type 2 diabetes (T2D), that are characterized by different combinations of cardiovascular (CV) risk factors, including dyslipidemia, abdominal obesity, hypertension, hyperglycemia/insulin resistance, and vascular inflammation. These risk factors individually and interdependently increase the risk of CV and cerebrovascular events, and represent one of the biggest health challenges worldwide today. CV diseases account for almost 50% of all deaths in Europe and around 30% of all deaths worldwide. Furthermore, the risk of CV death is increased twofold to fourfold in people with T2D. Whilst the clinical management of CV disease has improved in Western Europe, the pandemic of obesity and T2D reduces the impact of these gains. This, together with the growing, aging population, means the number of CV deaths is predicted to increase from 17.1 million worldwide in 2004 to 23.6 million in 2030. The recommended treatment for MetS is lifestyle change followed by treatment for the individual risk factors. Numerous studies have shown that lowering low-density lipoprotein-cholesterol (LDL-C) levels using statins can significantly reduce CV risk in people with and without T2D or MetS. However, the risk of major vascular events in those attaining the maximum levels of LDL-C-reduction is only reduced by around one-third, which leaves substantial residual risk. Recent studies suggest that low high-density lipoprotein-cholesterol (HDL-C) (<1 .0 mmol/l; 40 mg/dl) and high triglyceride levels (≥1.7 mmol/l; 150 mg/dl) are independent risk factors for CV disease and that the relationship between HDL-C and CV risk persists even when on-treatment LDL-C levels are low (<1.7 mmol/l; 70 mg/dl). European guidelines highlight the importance of reducing residual risk by targeting these risk factors in addition to LDL-C. This is particularly important in patients with T2D and MetS because obesity and high levels of glycated hemoglobin are directly related to low levels of HDL-C and high triglyceride. Although most statins have a similar low-density lipoprotein-lowering efficacy, differences in chemical structure and pharmacokinetic profile can lead to variations in pleiotropic effects (for example, high-density lipoprotein-elevating efficacy), adverse event profiles, and drug-drug interactions. The choice of statin should therefore depend on the needs of the individual patient. The following reviews will discuss the potential benefits of pitavastatin versus other statins in the treatment of patients with dyslipidemia and MetS or T2D, focusing on its effects on HDL-C quantity and quality, its potential impact on atherosclerosis and CV risk, and its metabolic characteristics that reduce the risk of drug interactions. Recent controversies surrounding the potentially diabetogenic effects of statins will also be discussed.

## Introduction

According to the World Health Organization, 63% of the 57 million deaths in 2008 were due to noncommunicable diseases [1]. Of these, cardiovascular (CV) diseases were the most common, accounting for approximately 30% of all deaths globally, followed by cancers (13%), chronic lung diseases (7%) and diabetes (2%). Whilst noncommunicable disease mortality rates have fallen in the developed world during recent years, rates continue to increase in lower income populations, with approximately 80% of all noncommunicable disease deaths occurring in low-income and middle-income countries. Of these deaths, 29% occur in people under the age of 60 years.

Of the 17.3 million CV deaths in 2008, 7.3 million were due to coronary heart disease and 6.2 million were due to stroke [2]. Major modifiable risk factors for CV disease and other noncommunicable diseases include hypertension, dyslipidemia, tobacco use, low fruit-vegetable intake, alcohol use, physical inactivity and high body mass index. Rather than existing in isolation, these risk factors tend to occur in clusters [3]. Data from the National Health and Nutrition Examination Survey (1999 to 2000) showed that 93.1%, 73.0%, and 35.9% of US adults had ≥1, ≥2, and ≥3 modifiable risk factors for CV disease, respectively [4]. Since each additional risk factor has a multiplicative, rather than an additive, effect on vascular risk [5], patients with clusters of risk factors have a significantly increased risk of developing CV and cerebrovascular disease. Metabolic syndrome (MetS), for example – characterized by three or more of the following: abdominal obesity, atherogenic dyslipidemia, hypertension, and/or insulin resistance with or without glucose intolerance [6-10] – is associated with a twofold to fourfold increased risk of stroke, a threefold to fourfold increased risk of myocardial infarction [11,12], and a fivefold to ninefold higher risk of developing type 2 diabetes (T2D) [13]. Similarly, T2D is associated with a twofold to fourfold increased risk of CV death [14]. Given that many CV risk factors are silent, patients with individual risk factors should be assessed for their overall cardiometabolic profile and treated accordingly.

The recommended treatment for MetS is lifestyle change, focusing on weight loss and physical activity, followed by pharmaceutical treatment for the individual risk factors [6,8-10]. Dyslipidemia – an imbalance between the proatherogenic apolipoprotein-B-containing lipoproteins (low-density lipoproteins, very-low density lipoproteins and chylomicrons/chylomicron remnants) and anti-atherogenic high-density lipoproteins (HDLs) – is a major risk factor for CV and cerebrovascular disease [5,15]. Numerous studies show that lowering low-density lipoprotein-cholesterol (LDL-C) levels using statins can significantly reduce CV risk in people with and without T2D, with no lower limit beyond which LDL-C-lowering is not beneficial [16-23]. Based on these results, most international treatment guidelines recommend lowering LDL-C to <2.6 mmol/l (100 mg/dl) in patients with established CV disease and to <1.8 to 2.0 mmol/l (70 to 80 mg/dl) in those with very high CV risk, reducing total cholesterol to <4.5 mmol/l (174 mg/dl) with an option of <4 mmol/l (154 mg/dl) if feasible [6,9,24-26]. Although most statins (including atorvastatin, simvastatin and pitavastatin) have similar effects on LDL-C levels [27-37], differences in chemical structure and pharmacokinetic profile can lead to variations in pleiotropic effects, adverse event profiles and drug–drug interactions. The choice of statin should therefore depend on the characteristics and needs of the individual patient.

Despite the widespread availability of effective lipid-lowering drugs, the prevalence of hypercholesterolemia varies considerably throughout Europe, ranging from 3 to 53% in men and from 4 to 40% in women, depending on the country [38]. In most populations, almost 50% of people treated with lipid-lowering drugs have a total cholesterol level >6.5 mmol/l (251 mg/dl), suggesting that greater efforts are needed to identify and adequately treat people with hypercholesterolemia. Moreover, even in patients that fully attain their total cholesterol/LDL-C targets, the risk of major vascular events is only reduced by around one-third [17], leaving substantial residual risk. The identification and treatment of residual risk factors is therefore essential for the effective management of CV disease.

## The importance of high-density lipoprotein for reducing residual risk

**Figure 1:**
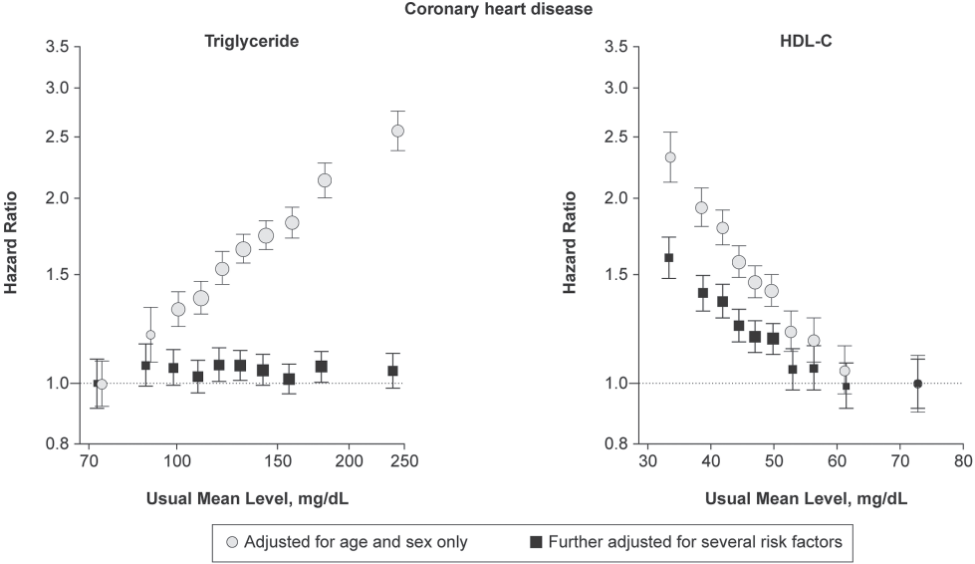
**High-density lipoprotein-cholesterol increase is associated with reduction in coronary heart disease risk, irrespective of triglyceride **[42]. HDL-C, high-density lipoprotein-cholesterol.

Numerous studies have shown that low levels of high-density lipoprotein-cholesterol (HDL-C) (defined as <1 mmol/l; 40 mg/dl in men, and <1.3 mmol/l; 50 mg/dl in women) are independent risk factors for coronary heart disease [39-46]. The Emerging Risk Factors Collaboration analyzed records from 68 studies in 302,430 people without initial vascular disease and demonstrated that each 0.38 mmol/l (14 mg/dl) increase in HDL-C level was associated with a 22% reduction in coronary heart disease risk, irrespective of the triglyceride (TG) level (Figure 1) [42]. Consequently, the recent European Society of Cardiology/European Atherosclerosis Society Guidelines for the Management of Dyslipidemia have included low HDL-C levels in their latest CV risk assessment charts [6,9,39]. This is particularly important for people with MetS or T2D who often have low levels of HDL-C accompanied by high levels of TG (≥1.7 mmol/l; 150 mg/dl) and a preponderance of small, dense low-density lipoprotein particles that can result in an underestimation of risk based solely on LDL-C. This triad of lipid abnormalities has been called atherogenic dyslipidemia [47-49].

A number of therapeutic options are available for increasing HDL-C levels, including statins, fibrates and niacin [6,39]. In general, niacin can increase HDL-C by 10 to 25%. However, recent results from the AIM-HIGH and HPS-2 THRIVE studies showed that the additional increases in HDL-C achieved when extended-release niacin was added to a statin did not result in further reductions in CV events [50,51]. Fibrates, on the contrary, can increase HDL levels by 2 to 10% and have been shown to reduce CV risk in people with significantly elevated levels of TG and reduced levels of HDL-C [52-55]. For example, the Action to Control Cardiovascular Risk in Diabetes (ACCORD) study found that the primary event rate (a composite of nonfatal myocardial infarction, stroke or CV death) was reduced from 17.3% to 12.4% in the subgroup of T2D patients with both low baseline levels of HDL-C (≤34 mg/dl or 0.88 mol/l) and high baseline TG (≥204 mg/dl or ≥2.3 mmol/l) [52]. However, this study also showed that the addition of fenofibrate to conventional statin treatment had no effect on event rates in people with normal levels of TG and/or HDL-C.

**Figure 2:**
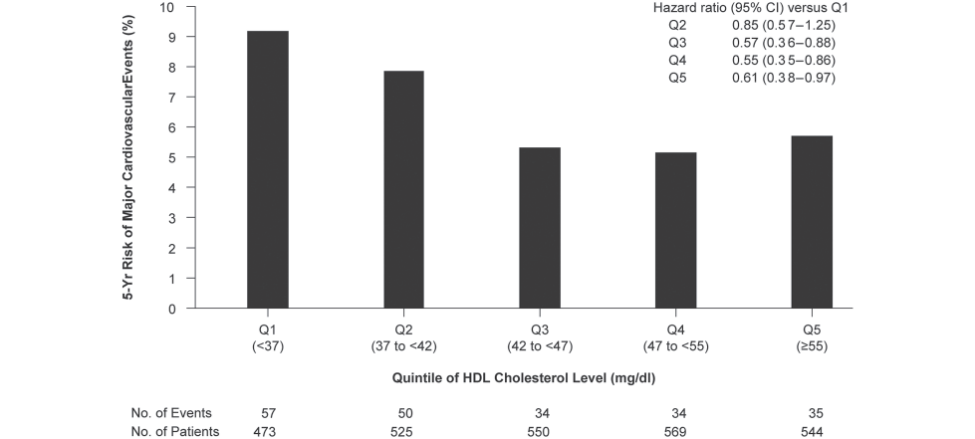
**High-density lipoprotein cholesterol predicts major cardiovascular events **[56]. CI, confidence interval; HDL, high-density lipoprotein; LDL-C, low-density lipoprotein-cholesterol.

To date, the only lipid-lowering studies in which drug-induced elevations in HDL-C have been found to correlate with reductions in CV risk involve statins [56-60]. A *post hoc* analysis of the Treating to New Targets (TNT) trial conducted in 2,661 subjects achieving LDL-C <1.8 mmol/l (70 mg/dl) during treatment with atorvastatin 10 or 80 mg/day showed that HDL-C levels were predictive of major CV events across the entire cohort, both when HDL-C was considered a continuous variable and when subjects were stratified according to HDL-C quintile [56]. This relationship remained true even after event rates were adjusted for other risk factors, including baseline levels of LDL-C (Figure 2).

Consistent with this observation, a *post hoc* analysis of intravascular ultrasound data from 1,455 people in four prospective randomized clinical trials showed that statin-associated changes in HDL-C were inversely associated with the progression of coronary atherosclerosis even in patients with low levels of LDL-C [61]. The proposed explanation for these findings is that statin-induced HDL elevations stimulate the 'reverse cholesterol transport' pathway, a process in which excess cholesterol is removed from peripheral cells and transported to the liver via HDL for excretion into bile [49]. Although most statins increase HDL-C levels to some extent, pitavastatin consistently produces significantly greater HDL elevations that are maintained, or increased, over time [29,58,60,62-64]. Pitavastatin is therefore likely to be particularly efficacious in people with low levels of HDL-C, such as those with cardiometabolic disease.

In addition to their role in cholesterol homeostasis, HDL particles have been shown to reduce oxidation, reduce vascular inflammation and vascular thrombosis, to improve endothelial function and repair, and to regulate the function and survival of pancreatic β-cells [47,49,65]. These functions are often defective in patients with inflammatory conditions such as MetS and T2D and can add to a patient's overall CV risk. Recent studies have shown that, in addition to elevating HDL levels, some lipid-lowering agents are associated with pleiotropic effects that improve HDL structure and function [47,49,66-68]. This observation is supported by a recent study, in which pitavastatin was associated with significantly greater reductions in plaque volume per 1% increase in HDL-C than other statins (atorvastatin, pravastatin, rosuvastatin, simvastatin) [67]. Future studies should therefore assess the effects of statins on HDL quality as well as quantity.

The following reviews will discuss the potential benefits of pitavastatin versus other statins in the treatment of patients with dyslipidemia, MetS or T2D, focusing on its effects on HDL-C quantity and quality, its impact on atherosclerosis and CV risk, and the avoidance of drug interactions. Recent controversies surrounding the potentially diabetogenic effects of statins will also be discussed, with a focus on the possibility that pitavastatin differs positively from other statins in this regard.

## Abbreviations

CV: cardiovascular; HDL: high-density lipoprotein; HDL-C: high-density lipoprotein-cholesterol; LDL-C: low-density lipoprotein-cholesterol; MetS: metabolic syndrome; T2D: type 2 diabetes; TG: triglyceride.

## Competing interests

HG is a consultant for Kowa.

## Declaration

This article has been published as part of *Cardiovascular Diabetology* Volume 12 Supplement 1, 2013: Statins in cardiometabolic disease: what makes pitavastatin different? The full contents of the supplement are available online at http://www.cardiab.com/supplements/12/S1. Publication of this supplement has been funded by Kowa Pharmaceutical Europe. Pitavastatin is a product marketed by the sponsor of the supplement. Articles are based on the proceedings of the World Congress for the Prevention of Diabetes. Authors received support with the preparation of their articles from GK Pharmacomm, an agency funded by the sponsor. All articles have undergone the journal's standard peer review process. The Supplement Editors declare that they have no competing interests.
